# History of Case 2

**Published:** 1915-09-25

**Authors:** 


### History of Case 2.

On May 8, the notes state, there was slight cardiac enlargement to the right of the sternum, and the apex beat was in the nipple line; no murmurs were audible. The tongue was very white and furred; the face was anæmic. The lungs offered no abnormal physical signs. The liver was just palpable below the costal margin; the spleen could not be felt. There was no abdominal tenderness nor rigidity. A copious, semi-morbilliform eruption was present on the limbs, trunk, face, and neck. The temperature was 105.8 deg. F. A sample of blood was taken; it was reported sterile, with a positive Widal reaction for *Bacillus typhosus*.

On May 12 I made the following note: "T. at the moment is normal. (This was in the morning.) Though still very anæmic, the patient is evidently not feeling acutely ill. Pulse rate 60. Both knee jerks very exaggerated. No Babinski reflex. Pseudo-clonus of ankles present. Kernig's sign indefinite. The tongue is still much furred. The rash is fading; no new crop has appeared since the last note. In places the erythematous patches contain a minute vesicle in the centre.

On May 18 the patient developed for the first time intense frontal headache, vomited once, and displayed some signs of cerebral irritation. The patient was very pale and anæmic, and very depressed about his condition. On May 21 a fresh crop of eruption appeared—not very plentiful, and chiefly on the abdomen. Frontal and occipital headache with vomiting and Cheyne Stokes breathing during the night. The pupils were normal: some ptosis of the left eyelid. The left disc was normal: the edges of the right disc were rather blurred, but there was no swelling or tortuosity of the veins. There was distinct stiffness of the neck: even passive flexion of the head was impossible.

On May 26 the neck was stiffer than before. Flexion was impossible, and efforts to produce it caused great pain. The headache was less severe than previously. The temperature was ranging from 101 to 102 deg., of a continued rather than intermittent type. On the abdomen and thighs was a large quantity of fine purpuric eruption. The ptosis of the left eye was unchanged. After this all symptoms vanished. On June 3 the knee jerks were but little brisker than normal, and speedy convalescence ensued. Lumbar puncture was not performed on this patient; but when towards the climax of his illness the symptoms of cerebral irritation developed I had the post-nasal fossa cultivated without any result: the same negative result followed this procedure on the English soldier, who was by that time convalescent.

It will be seen that the later symptoms of the French soldier pointed strongly to cerebro-spinal fever. Stiffness of the neck, ptosis, purpuric eruption, vomiting, intense headache, constituted a syndrome which was highly suspicious and suggestive. Up to that point his symptoms had been an exact copy of those displayed by the Englishman.

### Two Further Cases.

The next two cases are more indefinite still, and more difficult to diagnose. An English soldier, C. S., aged forty-two, reported sick on May 19 for backache and pains in the legs, which symptoms he had then had for one week. He had also lost his appetite, and had felt sick, but had not actually vomited. He arrived in a base hospital on May 27, by which time headache with pain and stiffness in the neck had supervened, with general weakness. For two days before his arrival he had had an eruption on his arms and legs. He had no cough nor nose-bleeding. He had sweated freely the day before admission. On admission the lungs and heart appeared normal. The abdomen was not distended; the spleen was not palpable. There was no rash on the trunk: the abdominal and cremasteric reflexes were absent. There was no *tache cérébrale*. A musty odour was perceptible. The knee jerks were normal; plantar reflexes were absent altogether. A modified Kernig's sign was present. The pupils were equal, and reacted both to light and to accommodation: there was no ocular palsy, nor any nystagmus. On the dorsum of each foot and on the extensor surface of each elbow an erythematous rash with an urticarial element was present. There were no diplococci found in a culture inoculated from the nasopharynx.

Lumbar puncture was immediately performed: clear fluid, not under unusual tension, was recovered. It was cultivated and found to be sterile. Next day the rash had all disappeared, and all symptoms were subsiding. On June 7 a note says: "No further developments. Patient complains much of pain in the back and legs, but there are no physical signs of anything abnormal." During the next two weeks this man had two rises of temperature for no obvious reason, each lasting twenty-four to forty-eight hours: no rashes accompanied these outbreaks of pyrexia. On June 23 the knee jerks were still exaggerated, and the man still complained of headache and backache: he was transferred to a hospital ship.

The remaining case was that of A. C., an English soldier of nineteen, who was in his usual health up to May 23, when redness and watering of the right eye began. On admission to a base hospital on May 28 he complained of nothing but the eye: had no pyrexia, no malaise, and a good appetite. On June 1 he for the first time complained of sore throat and headache, with pains and stiffness in the legs, the back of the neck, and to a lesser degree in the arms. He had one shivering fit that day. There was no vomiting, no cough, no nose-bleeding, no coryza.

On June 2 his condition was as follows: "Conjunctivitis nearly gone; eyes normal but for the remains of this. Face rather blotchy; some discrete erythematous blotches on the palate. No definite tonsillitis, no rhinorrhœa. On the legs and arms, especially on their extensor surfaces, is a scattered erythematous rash in very slightly raised papules. There is a good deal of the same rash on the back, and a little on the neck: there are two patches of it on the chest, but none on the abdomen. The tongue is furred. T. is 102°. Chest and heart present nothing abnormal. Knee jerks much exaggerated; no Babinski sign; no ankle clonus. A modified Kernig's sign is present. The abdomen is normal. There are no enlarged glands in the groins or elsewhere."

On the same day lumbar puncture was performed: a large quantity of clear fluid was obtained, under distinctly abnormal pressure. While the fluid was running the man complained much of headache. Culture of the fluid proved it to be sterile: some of it was injected into a guinea-pig, which, six weeks later, remained extremely well. The nasopharynx was also cultivated: no meningococci were found.

Next day the patient felt much better: the rash was practically gone, the temperature was normal, the knee jerks also normal, and there was neither stiffness nor pain in the neck and limbs. Two days later a relapse occurred. The temperature rose to 103°, the neck became distinctly stiff, the tongue very dirty, the knee jerks exaggerated, and a fresh crop of rash appeared. This rash was similar in character to the former crop, but less abundant: there was more of it on the legs than anywhere else. Two days later the man was much better: the rash had gone, the neck was no longer stiff, and the temperature was normal. Steady convalescence followed.

It is tempting to suppose that these two men were suffering from the same disorder as the first two, and that its rapid subsidence was due to prompt lumbar puncture, upon the great therapeutic value of which in cerebro-spinal fever most of the recent writers lay stress. Whether such a supposition is correct is impossible to say in the absence of all bacteriological foundations for such a slender clinical superstructure. And in fact the whole four cases are full of difficulties and problems. I am told that French army surgeons describe similar cases as occurring about the end of epidemics of cerebro-spinal fever when the virulence of the infection was becoming spent. Imperfect and fragmentary as the records of them are, I present them as the best which active-service conditions permitted me to make. My acquaintance with genuine cerebro-spinal fever of the classical and unmistakable type is confined to one case: so it is with diffidence and a full measure of scientific scepticism that I suggest the possibility of these four cases—and more especially the first two of them—being aberrant examples of the same malady.

